# Heat shock protein 27 regulates human prostate cancer cell motility and metastatic progression

**DOI:** 10.18632/oncotarget.1917

**Published:** 2014-04-21

**Authors:** Eric A Voll, Irene M Ogden, Janet M Pavese, XiaoKe Huang, Li Xu, Borko D Jovanovic, Raymond C Bergan

**Affiliations:** ^1^ Department of Medicine, Northwestern University, 303 E Superior, Chicago, IL; ^2^ Department of Preventive Medicine, Northwestern University, 303 E Superior, Chicago, IL; ^3^ Robert H. Lurie Cancer Center and Northwestern University, 303 E Superior, Chicago, IL; ^4^ Center for Molecular Innovation and Drug Discovery, Northwestern University, 303 E Superior, Chicago, IL

**Keywords:** Prostate Cancer, Metastasis, Heat Shock Protein 27, Matrix Metalloprotease-2, Cell Invasion

## Abstract

Prostate cancer (PCa) is the most common form of cancer in American men. Mortality from PCa is caused by the movement of cancer cells from the primary organ to form metastatic tumors at distant sites. Heat shock protein 27 (HSP27) is known to increase human PCa cell invasion and its overexpression is associated with metastatic disease. The role of HSP27 in driving PCa cell movement from the prostate to distant metastatic sites is unknown. Increased HSP27 expression increased metastasis as well as primary tumor mass. *In vitro* studies further examined the mechanism of HSP27-induced metastatic behavior. HSP27 did not affect cell detachment, adhesion, or migration, but did increase cell invasion. Cell invasion was dependent upon matrix metalloproteinase 2 (MMP-2), whose expression was increased by HSP27. *In vivo*, HSP27 induced commensurate changes in MMP-2 expression in tumors. These findings demonstrate that HSP27 drives metastatic spread of cancer cells from the prostate to distant sites, does so across a continuum of expression levels, and identifies HSP27-driven increases in MMP-2 expression as functionally relevant. These findings add to prior studies demonstrating that HSP27 increases PCa cell motility, growth and survival. Together, they demonstrate that HSP27 plays an important role in PCa progression.

## INTRODUCTION

Prostate cancer (PCa) is the second most common cause of cancer-related death and the most commonly diagnosed form of cancer for American males [1]. PCa mortality is caused by the movement of cancer cells from their point of origin within the prostate gland to multiple distant organ sites throughout the body [1]. Increased cell invasion constitutes a primary cellular characteristic of the metastatic phenotype, and this in turn stems from increased cell migration and/or increased protease activity [2].

Heat shock protein 27 (HSP27) expression has been associated with PCa progression, and in the development of metastasis in particular, in several clinical studies [3-6]. HSP27 expression is correlated with clinical PCa progression, with highest expression found in metastatic PCa [7-10]. Further, HSP27 was found to be an independent predictor of poor clinical outcome for PCa [6, 10].

We have previously shown that HSP27 increases human PCa cell invasion in human PCa cells, that this is mediated by upstream serial signaling through, MKK4 (MEK4), p38 MAPK and then MAPKAPK2, and that HSP27-mediated increases in invasion are dependent upon phosphorylation of HSP27 [11, 12]. Interestingly, unphosphorylated HSP27 has been shown to act as an actin capping protein, while phosphorylated HSP27 loses actin capping function, thus allowing actin polymerization [13-18]. Actin reorganization is a central component in regulating cell adhesion, detachment, motility and thus metastatic behavior [19].

Importantly, findings by several other groups demonstrate that HSP27 regulates functions in human PCa in addition to cell motility. HSP27 was shown to facilitate progression to androgen-independent PCa, in association with HSP27-mediated increase in AR transcriptional activity [7, 20]. HSP27 has been shown to enhance cell proliferation and suppress Fas-induced apoptosis in PCa cells [21]. More recently, HSP27 has been shown to regulate epithelial to mesenchymal transition (EMT) in PCa [22, 23]. OGX-427 targets HSP27 through an antisense mechanism, and is currently being tested in clinical trials in PCa (ClinicalTrials.gov #: NCT01120470).

The earliest steps in the metastatic cascade are cell detachment and cell invasion [24, 25]. The process of cell invasion involves increased cell migration coupled to proteolysis of the extracellular matrix (ECM) [24, 25]. The matrix metalloproteinases (MMPs) are a family of zinc-dependent endopeptidases that are secreted by tumor cells, and play a central role in cancer cell-mediated proteolysis. MMP-2, or gelatinase A, is an important MMP that is increased in several cancers, and in PCa in particular [26-29]. MMP-2 degrades collagen IV, which is a major constituent of the basement membrane in the prostate gland [30-32]. We have previously demonstrated that HSP27 increases MMP-2 expression in human PCa cells [11, 12]. Also, we have shown that inhibition MKK4, an upstream activator of HSP27, by small molecule therapeutics decreases MMP-2, inhibits cell invasion, and inhibits human PCa cell metastasis in pre-clinical studies [11, 12, 33]. Further, in prospective human clinical trials we have demonstrated that MKK4-targeting small molecule therapeutics will decrease MMP-2 expression in human prostate tissue [33].

Taken together, current findings demonstrate that increased HSP27 portends the future development of metastasis in humans, and *in vitro* studies implicate it in transformation to a high-motility metastatic phenotype. However, the role of HSP27 in driving the movement of PCa cells out of the prostate gland leading to the development of distant metastasis is currently unknown. Further, the mechanism by which HSP27 induces the invasive phenotype at the cellular level is not known.

In the current study, we demonstrate that HSP27 drives movement of human PCa cells out of the prostate gland to distant organs. Further, by examining a range of HSP27 expression levels, we demonstrate that HSP27's effect in this regard is proportional to its level of expression across a continuum of expression levels. Related studies demonstrated that differential HSP27 expression did not affect cell adhesion or cell detachment. Finally, we demonstrate that HSP27-mediated cell invasion is dependent upon MMP-2 expression.

## RESULTS

### Generation of HSP27 overexpression and knockdown variants

**Figure 1:**
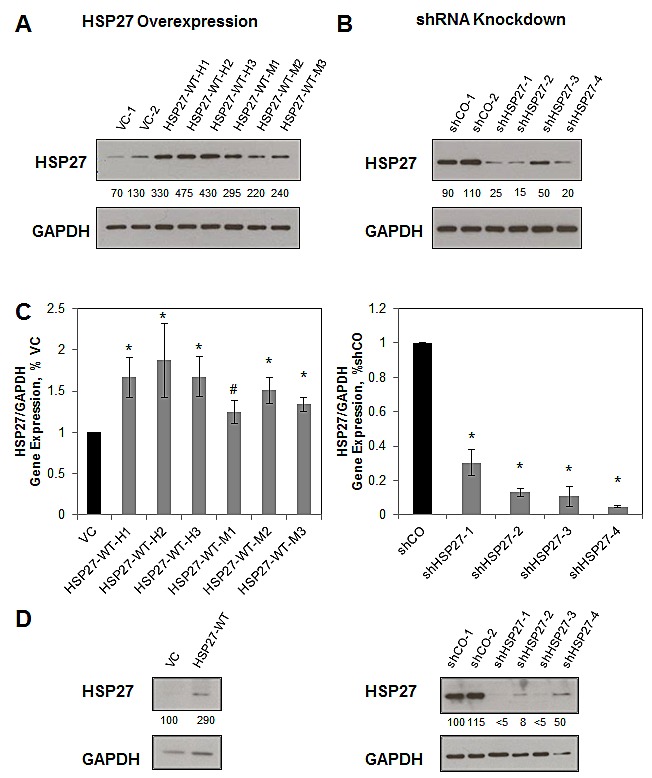
Establishment of HSP27 variant stable cell lines PC3-M cells were transfected with either wild-type HSP27 (HSP27-WT), empty vector control (VC), short hairpin RNA targeting HSP27 (shHSP27) or shRNA negative control (shCO), and resultant clonal cell lines selected as described in Methods. HSP27 protein expression was measured by Western blot in resultant overexpression and VC cell lines (A), or in shHSP27 and shCO cell lines (B). Depicted blots are representative, with similar results seen at least one separate experiment. Numbers indicate the mean band density calculated from at least two separate blots, expressed relative to the average of controls and normalized to GAPDH expression (C) HSP27 gene expression was measured by qRT/PCR, and was normalized to GAPDH and to VC or to shCO, as indicated. The mean value for VC1 and VC2 cells was normalized to 1.0, as was that for shCO1 and shCO2. Data represent the mean ± SEM from at least two independent experiments, each performed in replicates of N=2; *, p<0.05 by two-tailed student's t-test compared to controls; #, p<0.05 by one-tailed student's t-test compared to controls. (D) DU145 cells were transfected with either wild-type HSP27 (HSP27-WT), empty vector control (VC), short hairpin RNA targeting HSP27 (shHSP27) or shRNA negative control and HSP27 protein expression was measured by Western blot. Depicted blots are representative, with similar results seen at least one separate experiment. Numbers indicate the mean band density calculated from at least two separate blots, expressed relative to the average of controls and normalized to GAPDH expression.

To determine the role of HSP27 in regulating human prostate cancer (PCa) metastasis, we first generated a set of stable HSP27 variant cell lines. Individual over expression cell line variants were engineered by transfecting human PC3-M cells with wild-type HSP27 (HSP27-WT), and selecting individual emergent clones expressing high levels of HSP27. The associated control cell lines were similarly generated by transfection with empty vector (VC). HSP27 knockdown cell lines were created using short hairpin RNA targeting HSP27 (shHSP27), while the associated controls used non-targeting shRNA (shCO). Protein expression by individual cell lines was evaluated by Western blot, (Figs. 1A-B). Over expressing cell lines were sub-classified as moderate-level overexpression (HSP27-WT-M), if their level of HSP27 expression was between 200% and 300% of that of the average of vector control cells, and as high-level overexpression (HSP27-WT-H), if levels were above 300%. In knockdown cell lines, HSP27 protein levels were 50% or less, compared to the average of shCO cells. Each cell line was transfected with a single hairpin targeting the HSP27 gene, shHSP27-2 and shHSP27-3 had the same hairpin, while shHSP27-1 and shHSP27-4 each had different, unique, hairpins. Using qRT/PCR, we measured the expression of HSP27 transcript levels in each cell line (Fig 1C). In each instance, transcript levels were significantly altered, generally mirroring the observed changes in protein expression. We also generated pooled stable variants of DU145 PCa cells using the same constructs for HSP27 overexpression and knockdown, and confirmed differential HSP27 expression by western blot (Fig 1D). These findings demonstrate that it is possible to develop viable stable cell lines expressing either increased or decreased levels of HSP27, and that there is concordance between levels of gene and protein expression.

### Chronic changes in HSP27 expression regulate human prostate cancer cell invasion

**Figure 2:**
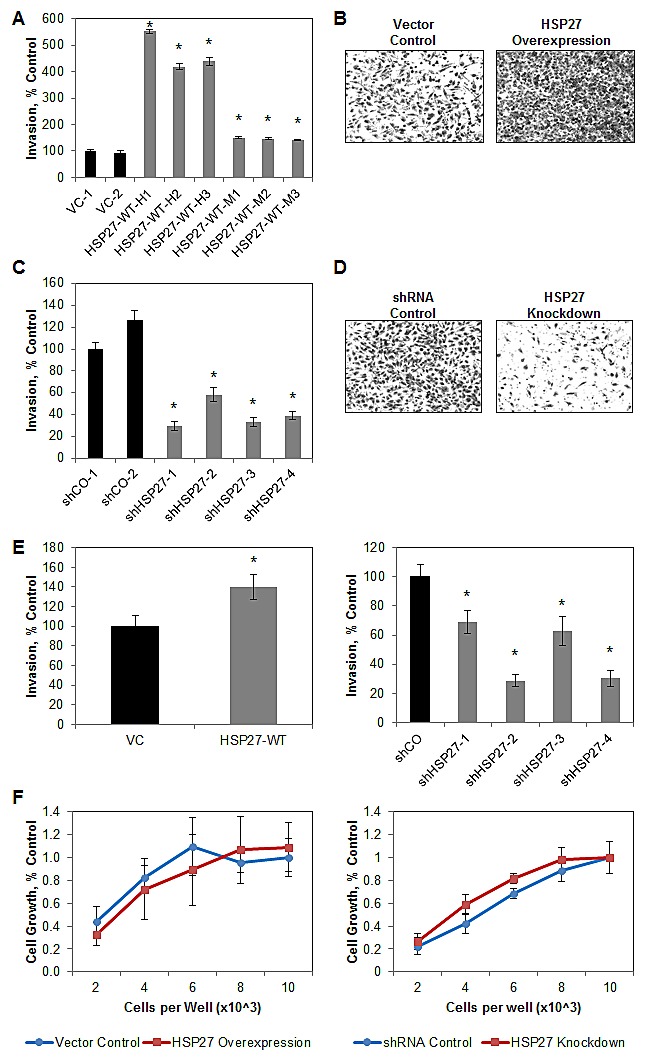
Sustained increases in HSP27 increase PCa cell invasion The effect of sustained HSP27 overexpression (A,B) or knockdown (C,D) on PC3-M cell invasion was assessed by Boyden chamber Matrigel invasion assay. Data represent the mean ± SEM of 2 independent experiments, each in replicates of N=3. * denotes p<0.05 compared to VC-1 or shCO-1. Representative images of control and overexpression (B) and of shCO and shHSP27 (D) cell lines, stained with crystal violet and imaged at 100X magnification, are depicted. (E) The effect of sustained HSP27 overexpression (left) or knockdown (right) on DU145 cell invasion was assessed by Boyden chamber Matrigel invasion assay. Data represent the mean ± SEM of a single representative experiment, in replicates of N=3, with similar results observed in multiple separate experiments. * denotes p<0.05 compared to VC or shCO. (F) The effect of HSP27 on cell growth. The indicated cell lines were plated at 2, 4, 6, 8, or 10 × 10^3^ cells per well in a 96-well plate, and OD_550_ determined after five days. Data represent the mean ± SEM of 2 vector control cell lines and 6 overexpression cell lines (left), or 2 shRNA control and 4 shRNA knockdown cell lines (right), from a single experiment run in replicates of N=2; similar results were observed in a separate experiment, also N=2.

Cell invasion is an early and critical step in the metastatic cascade [2, 24, 34]. We have previously demonstrated, under transient engineering conditions, that HSP27 will increase PCa cell invasion [11, 12]. However, the effect of sustained alterations in HSP27 expression is not known. As can be seen in Figs 2A and B, overexpression of HSP27 significantly increases invasion in all PC3-M-derived cell lines evaluated, compared to VC cells. Interestingly, our findings indicate that even in the context of overexpression, changes in the level of HSP27 expression affect the degree of invasion. Specifically, for the HSP27-WT-H subset of cell lines, expressing high levels of HSP27, mean cell invasion is approximately 450% of that of VC cells, while for the HSP27-WT-M, expressing moderately high levels of HSP27, mean cell invasion is only approximately 150% of that of VC cells. Conversely, knockdown of HSP27 significantly decreases cell invasion to a mean of approximately 30% of controls in all cell lines evaluated, Figs 2C and D. In order to corroborate these findings, we expanded studies to DU145 human PCa cells. Further, we did not expand our individual cell clones, but pooled all cells after antibiotic selection. Here too, HSP27 overexpression significantly increased cell invasion compared to controls, while HSP27 knockdown significantly decreased cell invasion in each of the shHSP27 constructs tested (Fig 2E).

We next examined the effect of HSP27 expression on the growth of individual PC3-M-derived cell lines. Figure 2F depicts representative data from a 5-day MTT assay, showing no significant change in cell growth for HSP27 overexpression compared to VC cells, or for HSP27 knockdown compared to shCO cells. Taken together, these findings demonstrate that chronic alterations in HSP27 expression impact the invasive phenotype of human PCa cells *in vitro*. Surprisingly, HSP27 expression did not affect cell growth in our experimental model system.

### HSP27 Regulates Human Prostate Cancer Metastasis

HSP27 expression is increased in primary PCa lesions [6, 10, 35], but its role in regulating their progression to distant metastasis remains unclear. We demonstrated above that chronic overexpression of HSP27 increases cell invasion *in vitro*. Given that cell invasion constitutes an initial step in the metastatic cascade, we hypothesized that HSP27 would increase invasion of cells out of the prostate gland and that this would result in increased formation of distant metastasis.

**Figure 3:**
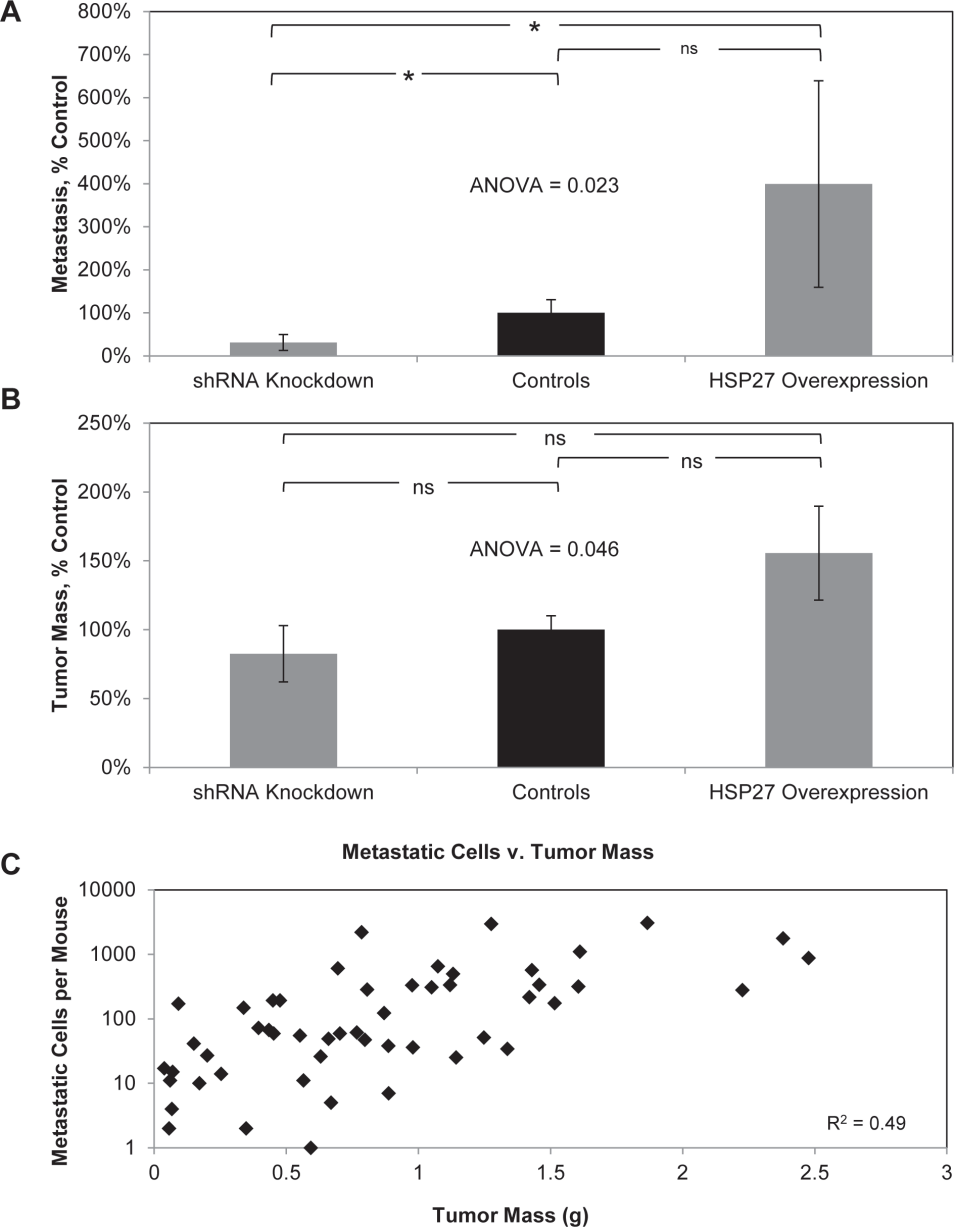
HSP27 increases PCa metastasis as well as tumor growth Cohorts of ten mice were orthotopically implanted with HSP27-WT-H1, HSP27-WT-H2, VC-1, VC-2, shHSP27-1, shHSP27-2, shCO-1 or shCO-2 cells. The resultant number of distant metastases (A) and tumor mass (B) were then measured. Data from HSP27-WT-H1 and HSP27-WT-H2 cohorts were combined, giving a HSP27 overexpression cohort (20 mice), that of VC-1, VC-2, shCO-1 and shCO-2 cohorts combined, giving a control cohort (40 mice) that was normalized to 100%, and that from shHSP27-1 and shHSP27-2 cohorts combined, giving a shRNA knockdown cohort (20 mice), Data are the mean ± SEM number of metastatic cells (A) and tumor mass (B) per mouse, normalized to controls. * denotes two-tailed Fisher's exact test p<0.05, ns denotes two-tailed Fisher's exact test p>0.05. (C) The graph depicts the number of metastatic cells plotted against the tumor mass for each mouse. The coefficient of determination (R^2^) between these two parameters was determined, and is depicted.

To test this, we employed an orthotopic murine model of human PCa metastasis originally developed by Stephenson et. al. [36] and further refined and characterized by us to allow for quantification of distant organ metastasis [37, 38]. This model closely recapitulates human disease in that it requires cells to complete all of the steps in the metastatic cascade, including initial steps, such as invasion out of the prostate gland, as well as latter steps, involving formation of distant metastasis. There were four different cohorts of mice, 20 mice per cohort, and they were implanted with cell lines as follows: vector control (10 mice implanted with VC-1 cells, 10 with VC-2 cells), HSP27 overexpression (10 mice with HSP27-WT-H1 cells, 10 with HSP27-WT-H2 cells), shRNA control (10 mice with shCO-1 cells, 10 with shCO-2 cells), and HSP27 knockdown (10 mice with shHSP27-1 cells, 10 mice with shHSP27-2 cells). Six weeks after implanting cells into the prostate of 6-8 week old male Balb/c athymic mice, distant metastasis was quantified. Overexpression and knockdown mice by necessity had different controls, based upon the fact that each required transfection with different vector constructs. In order to examine the effect of HSP27 upon metastasis, and to do so across a range of HSP27 expression, we normalized metastasis in knockdown and overexpression mice to their relevant controls. It can be seen in Fig. 3A that the number of metastases in knockdown mice was 30% of that of controls, while in overexpression mice it was 400% of controls, ANOVA p value = 0.023. Comparisons between individual groups were also performed. We did so by determining the median value of metastasis in control mice, and then used Fisher's exact test to evaluate whether mice in the other groups had more or less metastasis than the median value. In this manner, knockdown mice were shown to have significantly less metastasis than controls (2-sided p value 0.03), overexpression mice exhibited a trend towards an increase compared to control (p value 0.28), and overexpression mice had significantly more metastasis compared to knockdown mice (p value 0.005). These findings demonstrate for the first time that increased HSP27 expression in primary tumors will increase the formation of distant metastases, and that this effect is observed across a spectrum of HSP27 expression levels.

We also measured primary prostate tumor size in each mouse, Fig. 3B. In HSP27 overexpressing mice, the tumor mass was 225% of that of control mice, while with knockdown mice it was 30% of controls. Considered across all three cohorts, HSP27 significantly increased tumor mass, ANOVA p value = 0.046. For comparisons between groups, tumors in overexpression mice were non-significantly higher than both control mice (Fisher's exact test 2-sided p value 1.0) and knockdown mice (p = 0.44), and those of knockdown mice were non-significantly lower than that of controls (p = 0.36). These findings demonstrate that HSP27 expression regulates tumor growth. Given that HSP27 did not affect cell growth *in vitro*, they also demonstrate that the outcome *in vivo* is not a foregone conclusion based upon *in vitro* analysis.

It is recognized that the propensity to metastasize increases as tumor mass increases. This raises the possibility that the differences in metastasis observed in the current study are simply a function of differences in tumor mass. If this were the case, then tumor mass should closely correlate with number of metastasis. We therefore went on to determine the coefficient of determination (R^2^) for tumor mass versus number of metastasis at the individual mouse level, Fig 3C. There was a low correlation (R^2^ = 0.49) between tumor mass and metastasis. These findings demonstrate that HSP27 increases human PCa metastasis in addition to tumor growth. Of importance, both increased tumor mass and increased metastasis with HSP27 expression in the current study reflect findings from human studies wherein increases in HSP27 have been associated with more advanced stages and the development of metastasis.

### HSP27 increases cell proliferation *in vivo*

**Figure 4:**
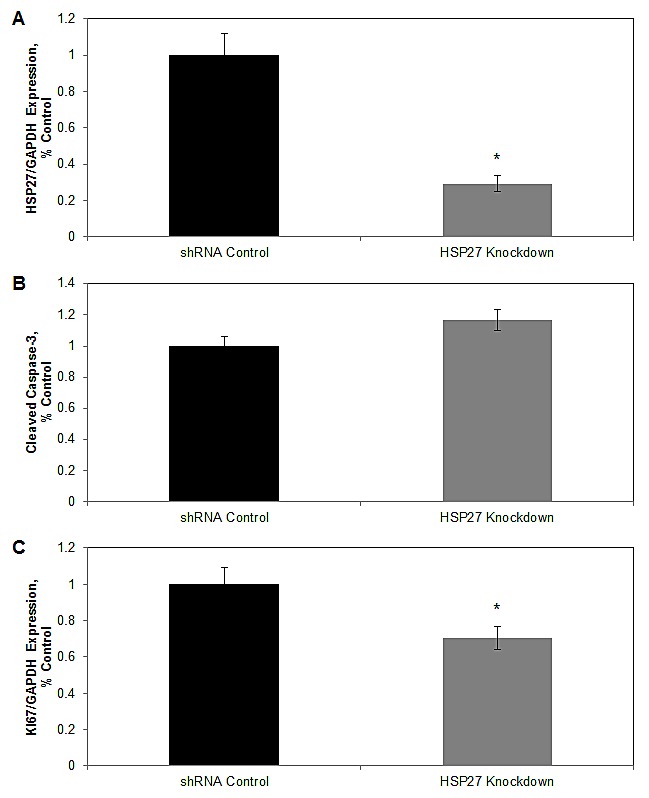
HSP27 increases cell proliferation *in vivo* Fresh frozen tumor tissue from shRNA control and shHSP27 knockdown mice was analyzed for expression of HSP27 by qRT/PCR (A), cleaved caspase-3 by ELISA (B), or for expression of Ki-67 (C) by qRT/PCR. Data represent mean ± SEM for all mice in a given cohort. The cleaved caspase-3 concentration was measured in each tumor, in replicates of N=2 per mouse. Gene expression, normalized to GAPDH, was assessed in at least 2 independent experiments, each in replicates of N=2. All respective control values were normalized to 1.0. * denotes Student's t-test p<0.05 compared to controls.

We conducted a series of experiments to examine molecular mechanisms operative within tumors to explain the observed increase in tumor mass. At the time of implantation, overexpression and knockdown cells exhibited clear and significant increases and decreases in HSP27 expression, respectively (see Fig. 1). However, post-implantation cells were not under antibiotic selection. To assess whether expression changed with time, we measured HSP27 expression in tumors of each mouse. As can be seen in Fig. 4A, in knockdown mice HSP27 levels remained significantly suppressed. However, in overexpression mice HSP27 expression had returned back to its baseline level over the six week growth period in mice, in the absence antibiotic selection, and was not significantly different from that of controls (Fig. S1). Therefore, when interpreting results from tissue-based biomarkers, we considered findings from knockdown mice informative, while we considered those from overexpression mice not informative. This was based upon the assumption that biomarkers indicative of HSP27 action would be dependent upon ongoing HSP27 expression.

We first examined whether there were differences in apoptosis in the tumor samples, as determined by differences in cleaved caspase 3 protein levels, measured by ELISA. There were no significant differences in cleaved caspase 3 levels between tumors from HSP27 knockdown mice and their relevant controls (Fig. 4B).

We next examined potential differences in cell proliferation by measuring changes in gene expression of the cell proliferation marker, Ki-67, by gene-specific qRT-PCR, Fig 4C. Importantly, in informative knockdown mice, Ki67 levels were significantly reduced by 30% compared to controls. In overexpression mice Ki67 levels were unaltered, consistent with normalization of HSP27 expression with the associated control cohort (Fig. S1). These findings indicate that *in vivo* HSP27 increases PCa cell proliferation, but does not affect cell death. HSP27-induced increases in cell proliferation provide a rational explanation for the increased tumor size seen with increased HSP27.

Finally, others have shown that HSP27 induces epithelial-to-mesenchymal transition (EMT) in PCa cells, and have correlated this with increases in cell invasion as well as with increased cell growth [22]. A decrease in E-cadherin and an increase in vimentin are seen with EMT, and we therefore examined these markers in tumors. There was no significant difference in the protein levels of either E-cadherin or vimentin in tumors in either HSP27 over expressing or in HSP27 knockdown mice, compared to their respective controls (Fig. S2).

We went on to examine E-cadherin and vimentin expression in HSP27 over expressing and knockdown cell lines, and did so for both PC3-M and DU145 cell lines (Fig. S3). HSP27 did not exhibit a uniform effect across cell lines. Specifically, in the case of PC3-M cells, HSP27 over expression did in fact significantly decrease E-cadherin expression. While a concordant effect was not observed in over expression tumors, it is important to note that HSP27 over expression was lost with time in mice, and thus findings in these tumors were considered non-informative. In HSP27 over expressing PC3-M cells, vimentin was unchanged. In HSP27 knockdown cells, vimentin and E-cadherin were unchanged. In the case of DU145 cells, HSP27 over expression did not alter E-cadherin or vimentin. However, HSP27 knockdown increased E-cadherin and decreased vimentin. These findings demonstrate that different effects upon EMT are observed across cell lines, and as a function of whether HSP27 is increased or decreased.

### HSP27 does not affect PCa adhesion or detachment

**Figure 5:**
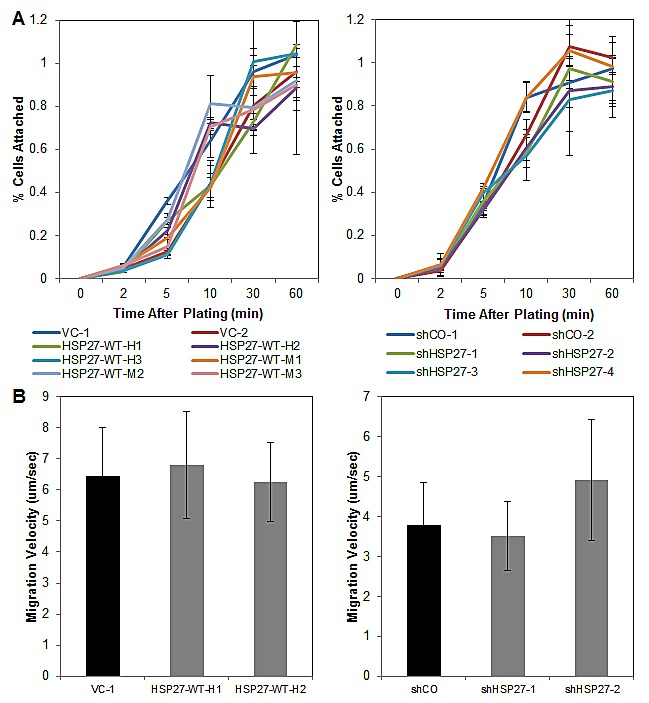
HSP27 does not affect cell adhesion nor cell migration (A) HSP27 expression does not affect PCa cell attachment. Cell attachment assays were performed as described in Methods. Data are the mean ± SEM attached cells with time after plating of the indicated overexpression cell lines (left) or shRNA knockdown cell lines (right). Data is from a single experiment performed in replicates of N=3, with similar results seen in multiple separate experiments, also N=3. (B) HSP27 expression does not affect cell migration. Single cell migration assays were performed as described in Methods. Data are the mean ± SEM cell velocity from a single experiment of N=35 individual cells, with similar findings seen in a separate experiment, of N>30 cells.

Having demonstrated that HSP27 is an important regulator of metastatic behavior, it became important to understand the cellular mechanisms underlying that behavior. Because HSP27 has known actin-capping function, we hypothesized that it would regulate cell attachment. We investigated this by performing cell detachment as well as cell adhesion assays. Surprisingly, we did not identify any effect of altered HSP27 expression upon either of these cellular processes. Depicted in Fig. 5A are representative findings of cell adhesion assays. The depicted data compare cell adhesion of HSP27 overexpression to VC cells, as well as HSP27 knockdown to shCO cells, on collagen I treated culture plates. Similarly negative findings were observed for cell adhesion assays under conditions involving coating of tissue culture plates with fibronectin and matrigel, as well as non-coated tissue culture plastic, and for cell detachment assays (Figs. S4-S5). These findings demonstrate that despite its actin-capping function, HSP27 did not regulate cell attachment or cell detachment in PC3-M cells.

### HSP27 does not affect PCa cell motility

We demonstrate in Fig. 2 that HSP27 regulates cell invasion. Cell invasion is a composite function, dependent upon the processes of kinetic changes in cell adhesion, cell attachment, cell migration, and proteolysis of the extracellular matrix. As our above findings demonstrate that HSP27 did not affect cell attachment or detachment, we next focused our investigations upon cell migration and protease action. To assess whether HSP27 regulates cell migration in human PCa cells, we performed single cell motility assays. With this assay, the movement of over 30 individual cells on collagen I coated plates are tracked with time-lapse imaging over 4.5 hours. As can be seen in Fig 5B, HSP27 did not affect cell velocity in all cell lines tested. Further, it did not affect distance travelled or changes in directionality (data not shown). These findings demonstrate that HSP27 does not affect cell migration in PC3-M cells.

### HSP27-driven cell invasion is abrogated by inhibition of MMP-2

**Figure 6:**
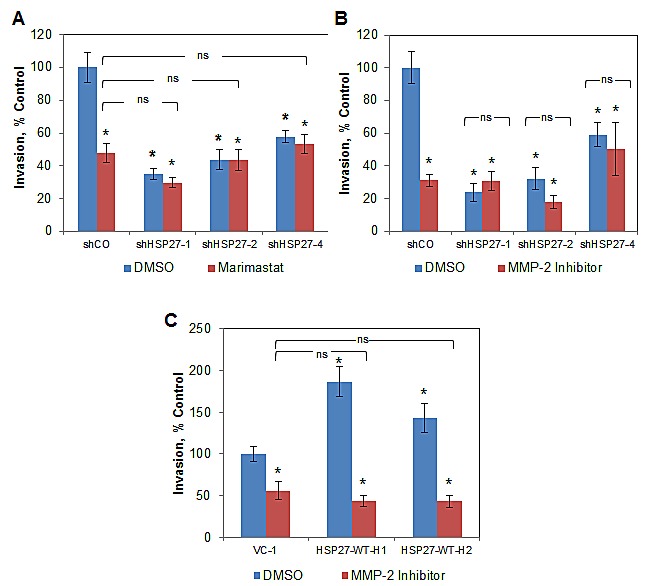
HSP27-driven cell invasion is dependent upon MMP-2 (A) Boyden chamber invasion assays were performed with treatment with either 10 μM Marimastat or DMSO. Data show mean invasion ± SEM of a single experiment, normalized to DMSO-treated shCO cells, performed in replicates of N=3, similar results were observed in a replicate experiment, also N=3. * denotes Student's t-test p<0.05 compared to DMSO-treated shCO. Matrigel Boyden chamber invasion assays were performed with HSP27 knockdown (B) or overexpression (C), treated with either 1 μM MMP-2 chemical inhibitor (*Cis*-9-octadecenoyl-N-hydroxylamide oleoyl-N-hydroxylamide) or DMSO. Data are the mean + SEM invasive cells of a single experiment, normalized to DMSO-treated controls, performed in replicates of N=3. Similar results were observed in separate experiments, also N=3. * denotes Student's t-test p<0.05 compared to DMSO-treated shCO.

We next examined the role of proteases in mediating HSP27 efficacy. Matrix metalloproteinases (MMPs) play major roles in degrading extracellular matrix proteins, including those of the basement membrane, thereby facilitating cell invasion and metastasis, and have been widely implicated in this role across many cancer types, including PCa [39-41]. To assess whether HSP27 efficacy was mediated through MMP activity, we first used the broad-spectrum MMP inhibitor, Marimastat. As shown in Fig. 6A, Marimastat significantly decreased cell invasion in control cells by over 50%. Importantly, knockdown of HSP27 did not further inhibit invasion in the face of Marimastat treatment, and HSP27 knockdown alone exerted a similar level of invasion inhibition as did Marimastat alone. This finding demonstrates that HSP27-driven cell invasion is dependent upon MMPs.

Related studies examined Marimastat in HSP27 over expressing cells (Fig. S6), demonstrating that Marimastat decreased the invasion of over expressing and control cells with similar efficacy. Whereas removal of a drug target will, by definition, negate drug efficacy, over expression of a drug target is much less informative. This is because over expression can decrease, have no effect, or increase drug efficacy, dependent upon the specifics of the particular system. Therefore, we considered HSP27 over expression findings with Marimastat mechanistically uninformative.

**Figure 7:**
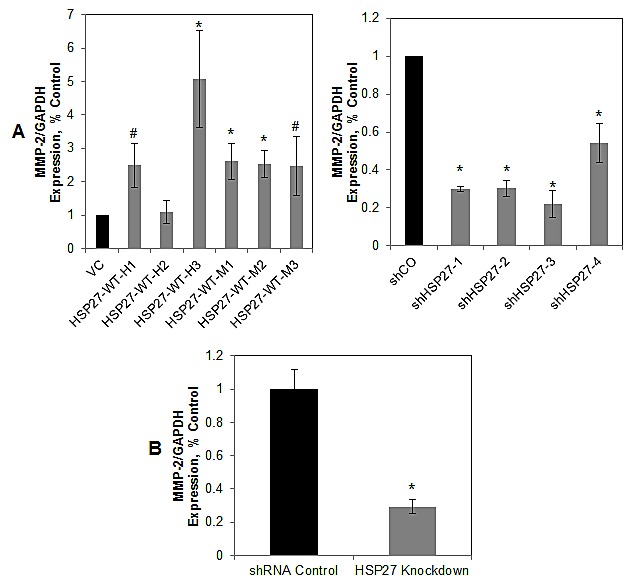
HSP27 regulates MMP-2 gene expression (A) HSP27 regulates MMP-2 gene expression *in vitro*. The expression of MMP-2 in HSP27 stable clones was measured by qRT/PCR normalized to GAPDH, as in Figure 1C, and expressed relative to control clones (normalized to 1.0). Data represent the mean ± SEM from at least two independent experiments performed in replicates of N=2; *, p<0.05 by two-tailed student's t-test compared to controls; #,p<0.05 by one-tailed student's t-test compared to controls. (B) HSP27 regulates MMP-2 gene expression *in vivo*. MMP-2 gene expression levels in mouse prostate tumor samples was measured as in (A). Data represents average MMP-2 gene expression ± SEM of at least 2 independent experiments, normalized to GAPDH, and expressed relative to shRNA control cells normalized to 1.0. *, p<0.05 compared to controls.

Because Marimastat is not MMP subtype specific, we explored specific MMPs. MMP-2 degrades collagen IV, which is a predominant component of the prostate basement membrane [30-32]. Increased MMP-2 in primary prostate tumors predicts the future development of metastatic disease in human studies [42, 43]. While our group, as well as others, have previously associated increases in HSP27 with that of MMP-2 [11, 22], it is not known whether MMP-2 mediates HSP27 function. We examined this by using the MMP-2 specific inhibitor, *Cis*-9-octadecenoyl-N-hydroxylamide oleoyl-N-hydroxylamide or DMSO (for non-treatment controls). As shown in Fig. 6B, inhibition of MMP-2 significantly decreased cell invasion by over 70% in control cells. Further, HSP27 knockdown had a similar effect, and importantly, MMP-2 inhibition did not further decrease invasion in the face of HSP27 knockdown. Finally, similar effects were seen in multiple cell lines tested. We next examined the effect of MMP-2 inhibition on cell invasion in HSP27 overexpressing cells (Fig. 6C). MMP-2 inhibition decreased invasion of control cells by almost 50%. Importantly, this effect was proportionally greater in HSP27 overexpressing cells. In fact, even though HSP27 overexpressing cells were much more invasive than controls, inhibition of MMP-2 decreased invasion to levels observed in control cells treated with MMP-2 inhibitor. In separate studies we also attempted to specifically knockdown MMP-2 using siRNA. However, because it was not possible to achieve knockdown levels of greater than 50% without losing specificity (data not shown), we did not pursue this further. These findings demonstrate for the first time that HSP27-mediated increases in cell invasion are specifically mediated by and dependent upon MMP-2. We next examined MMP-2 expression in the HSP27 variant cell lines and in tumors by qRT-PCR, Fig. 7. In PC3-M-derived cells MMP-2 gene expression was significantly increased in five of six HSP27 overexpression cell lines, and significantly decreased in all four HSP27 knockdown cell lines evaluated, Fig. 7A. In DU145 cells, basal levels of MMP-2 expression were too low to permit accurate analysis. In mouse tumor samples, MMP-2 was significantly decreased in informative knockdown mice, compared to relevant controls, Fig. 7B. In overexpression mice (where HSP27 levels had reverted back to those of control cells), MMP-2 levels were not significantly different (Fig. S1). Taken together, these findings demonstrate that HSP27-driven transformation to a metastatic phenotype in human PCa is dependent upon MMP-2.

## DISCUSSION

We have demonstrated for the first time that increased HSP27 expression in primary tumors drives PCa cell motility and resultant metastasis development. These findings complement our prior findings demonstrating that HSP27 will increase human PCa cell invasion. While other studies have examined the effect of HSP27 upon growth and survival of experimentally implanted metastasis, the current study demonstrates for the first time that HSP27 increases the development of metastasis originating from a primary tumor. This finding builds upon our prior demonstration that HSP27 increases PCa cell invasion. In addition, we demonstrate for the first time that HSP27-mediated cell invasion is dependent upon MMP-2. This builds upon our prior demonstration that HSP27 expression is associated with MMP-2 expression in human PCa [11, 12].

Several studies from different investigators, including our group, highlight the biological importance of HSP27 in human PCa progression. The primary focus of other investigators has been upon HSP27-mediated regulation of growth and survival (reviewed in [35]), while that of our group has been on HSP27-mediated regulation of PCa cell motility (reviewed in [44]). Together, studies by us and others all point to the importance of HSP27 in fostering human PCa progression, and serve to provide a complementary description of its several biological functions. We have previously reported in a prospective phase II randomized trial that small molecule therapeutics targeting MKK4, an upstream activator of HSP27, will decrease MMP-2 expression in human prostate tissue [33]. Our current findings lend further credence to the notion that this strategy provides a rational avenue for therapeutically inhibiting the development of PCa metastasis in humans. In addition, our findings provide direct support for the use of other HSP27-targeting therapeutic strategies in the setting of localized PCa, with the clinical goal of similarly inhibiting the development of metastasis. OGX-427, which is now undergoing phase II testing in the setting of metastatic PCa (ClinicalTrials.gov #: NCT01120470), is one such example.

In animals, increases in HSP27 were associated with both increased tumor growth and increased cell proliferation, as measured by Ki67 labeling. However, effects upon cell growth *in vitro* were not detected, despite extensive studies designed to detect such. Interestingly, our findings appear to differ from others who report that HSP27 increases cell growth *in vitro* [8, 21, 45, 46]. There are several potential explanations for this. The current study examined chronic alterations in HSP27 expression, while others primarily examine transient differences. In addition, less definable factors that relate to differences between laboratories may be responsible, and are not rare occurrences in the field of cancer biology. While exact causes fail to be identified in the majority of situations, differences in serum, which itself is not defined, are considered an important factor. In fact, our findings that HSP27 affects cell growth in animals, but not *in vitro*, indicates the importance of external factors in affecting outcome in the case of HSP27.

We found that EMT markers as a function of HSP27 expression revealed differences between cell lines. It is very likely that the same factors discussed above were contributory to differences between these variable findings across cell lines, and the constant ones reported by others. Such a situation is exactly what one would expect with chronic alterations in HSP27 expression, as was undertaken in the current study. Under circumstances of chronic perturbation, cells have the opportunity to react through a variety of different compensatory pathways.

It is important to note that the experiments in this study were purposefully carried out in androgen-independent PC3-M and DU145 PCa cell lines. Androgen signaling in PCa plays a central role, but is exquisitely complex. It will therefore be important for future investigations to examine how the androgen signaling axis interfaces with HSP27-mediated regulation of invasion and metastasis. It is already known that HSP27 facilitates the development of androgen-independence [7, 20]. Further, it is recognized that with time all PCa progresses to androgen-independence, particularly in the setting of metastasis. We therefore sought to examine the role of HSP27 in regulating metastatic behavior in the androgen-independent setting in the current study.

We had hypothesized that HSP27 would modulate cell adhesion and cell attachment, and found that it did not. Our hypothesis was based upon the facts that HSP27 is a known actin capping protein [13-18], that actin plays a central role in the regulation of cell detachment and adhesion, that changes in cell detachment and adhesion constitute early steps in the metastatic cascade and are integral to cell invasion [34], and that HSP27 is known to increase cell invasion [11]. It is important to consider that our findings do not rule out the possibility that HSP27-mediated actin capping function is necessary for cell motility. However, they do not support it, and suggest otherwise. In this regard, it is important to consider that we did go on to demonstrate that HSP27-mediated invasion is dependent upon MMP-2 expression. This finding serves to provide an alternative regulatory pathway, other than that related to actin capping. While the current study focused upon examining MMP-2 expression at the transcript level, we have previously demonstrated that HSP27-mediated changes in MMP-2 transcript lead to concomitant changes in MMP-2 function, as measured by zymography [11, 12].

## CONCLUSIONS

In this study, we demonstrate for the first time that HSP27 increases the movement of human PCa cells out of the prostate gland resulting in the formation of distant metastasis. We also demonstrate that HSP27-mediated increases in cell invasion are dependent upon MMP-2 expression. Our findings complement those of other groups who demonstrate that HSP27 increases human PCa cell growth and viability. Together, they demonstrate that HSP27 contributes to human PCa progression through a variety of cellular processes. Together, they serve to provide mechanistic explanations for clinical studies which associate high levels of HSP27 expression with the development of metastatic disease and poor prognosis in men with PCa.

## METHODS

### Cell Culture & Transfection

The origin and culture conditions of PC3-M and DU145 human PCa cells were previously described by us [38, 47]. Cells were maintained in RPMI 1640 (PC3-M) or DMEM (DU145) media supplemented with 2 mM L-glutamine, 10 mM HEPES buffer, and 10% fetal bovine serum (Life Technologies, Grand Island NY). Cells were incubated in a humidified atmosphere of 5% carbon dioxide at 37°C under sub-confluent exponential growth conditions, routinely tested for mycoplasma, routinely replenished from low passage stocks, and their morphology was confirmed at each passage under light microscopy.

Stable transfection was performed as follows: Cells were seeded in 6 well tissue culture plates at 2.5 × 10^4^ cells per well and incubated for 24 hours at 37°C in 5% carbon dioxide. After 24 hours, cells were transfected with 0.5 μg of plasmid and 5 μL TransIT-LT1 Transfection Reagent (Mirus Bio LLC, Madison, WI), per manufacturer's instructions. After 24 hours, media was replaced with RPMI supplemented as above, containing 500 μg/μL G418 Sulfate (Cellgro, Manassas, VA) for HSP27 overexpression vectors or 2 ng/μL puromycin (Sigma) for shRNA vectors. Cells remained under continued antibiotic selection conditions for approximately 1-2 additional weeks. PC3-M stable clones were then selected by western blot for HSP27 protein expression levels compared to non-transfected PC3-M, DU145 transfected cells were pooled and analyzed for HSP27 protein levels by western blot.

### Plasmids

HSP27 wild type pcDNA3.1-HSP27WT plasmids were kindly provided by Rainer Benndorf (University of Michigan, Ann Arbor, MI) and have been described previously [48]. HSP27 knockdown pLKO-PURO.1 lentiviral MISSION shRNA plasmids were obtained from Sigma (St. Louis). The hairpin sequences are as follows: MISSION pLKO.1-puro Non-Mammalian shRNA Control (shCO-1 and -2): CCGGCAACAAGATGAAGAGCACCAACTCGAGTTGGTGCTCTTCATTGTTGTTTTT, shHSP27-1: CCGGCCCGGACGAGCTGACGGTCAACTCGAGTTGACCGTCAGCTCGTCCGGGTTTTT, shHSP27-2 and -3: CCGGGATCACCATCCCAGTCACCTTCTCGAGAAGGTGACTGGGATGGTGATCTTTTT, and shHSP27-4: CCGGCCGATGAGACTGCCGCCAA GTCTCGAGACTTGGCGGCAGTCTCATCGGTTTTT.

### Western Blot

Western blotting was performed as previously described by us [49]. Briefly, cell lysates were centrifuged for 30 minutes at >10,000 rpm at 4°C. Protein concentration was measured using the Bradford method (Bio-Rad, Hercules, CA). Samples were separated by SDS-PAGE under reducing conditions and transferred to 0.45 μm nitrocellulose membrane (Bio-Rad, Hercules, CA), blocked with 5% milk in TBS-T (25 mM Tris-HCl, pH 7.4, 150 mM NaCl, 0.1% Tween 20), and probed with primary and secondary antibody per manufacturer's instructions. Membranes were stripped with 62.5 mM Tris (pH 6.8), 2% SDS, and 100 mM β-mercaptoethanol for 40 minutes at 50°C, blocked for 1 hour at room temperature, and re-probed as described above.

### Antibodies

HSP27 (G31) mouse monoclonal antibody and GAPDH (14C10), E-cadherin (24E10), and vimentin (D21H3) rabbit monoclonal antibodies were obtained from Cell Signaling Technology (Danvers, MA). ECL Anti-Rabbit IgG, Horseradish Peroxidase Linked whole antibody and ECL Anti-Mouse IgG, Horseradish Peroxidase Linked whole antibody were obtained from GE Healthcare UK (Buckinghamshire, UK)

### Quantitative Real-Time PCR

To measure gene expression, RNA was first isolated from cells with an RNEasy Mini Kit (QIAGEN, Valencia, CA), or from snap-frozen prostate tumor samples with Trizol (Invitrogen) followed by purification with RNEasy Mini Kit, both per manufacturer's instructions. The concentration and purity of isolated RNA was determined by Nanodrop (Thermo Scientific, Hanover Park, IL). cDNA was then synthesized using 1 μg of isolated RNA with TaqMan Reverse Transcription Reagents (Life Technologies) per manufacturer's instructions. Quantitative real-time PCR was carried out with TaqMan Universal PCR Master Mix and TaqMan primer-probe pairs on a 7500 Real Time PCR System (Life Technologies), as previously described by us [37]. Gene-specific exon-spanning primer/probe sets for HSP27, MMP-2, KI67 and GAPDH were obtained from Applied Biosystems (Foster City, CA). Each sample was run in duplicate and the mean threshold cycles (Ct) were used to calculate relative gene expression. Gene expression was normalized to GAPDH for each sample, and relative gene expression was computed using the 2^–ΔΔCt^ method [50]. All experiments were repeated at least once at a separate time.

### Orthotopic Mouse Model

Orthotopic implantation of human PCa cells was performed as previously described by us [38]. Six-to-eight week old male, athymic, balb/c mice were obtained from Charles River (Wilmington, MA) and treated under a Northwestern University approved IACUC protocol. Mice were housed in a barrier facility with 12 hour light/dark cycles, fed a diet of soy free chow (Harlan, Indianapolis, IN), and water *ad libitum*. For each mouse, 2.5 × 10^5^ cells were suspended in 0.9% Sodium Chloride (Hospira, Lake Forest, IL). Mice were anesthetized and cells were injected into the ventral lobe of the prostate. Six weeks after injection, mice were sacrificed. To detect metastases, formalin-fixed paraffin-embedded lung tissues were step sectioned in intervals of 45 μm, with three consecutive sections (4 μm thick) cut at each interval, up to a total of 15 sections per lung. Sections were mounted on charged plus slides and stained with hematoxylin and eosin and metastases were scored in a blinded fashion.

### ELISA

To measure the extent of apoptosis in each tumor, we performed a cleaved caspase-3 ELISA. Prostate tumors were first lysed using the protein lysis buffer described above and a tissue homogenizer. Total protein concentration was quantified using the Bradford method. Cleaved caspase-3 concentration was detected using the Invitrogen human caspase-3 (active) ELISA kit (Camarilo, CA), per manufacturer's instructions, with 250 μg total protein added to each well.

### Invasion Assays

Cells were suspended in serum-free RPMI or DMEM media containing 0.1% BSA and added to 8.0 μm pore Growth Factor-Reduced Matrigel Invasion Chambers (BD Biosciences) at 10^5^ cells/μL in four wells per experimental condition. NIH-3T3 conditioned media was used in the lower chamber of the invasion plate as a chemoattractant. After 24 hours, each well was washed with PBS, and cells on top of the membrane were removed with cotton-tipped applicators (Puritan Medical Products, Guilford, ME). Invaded cells were stained by submerging each well in a solution of 0.5% crystal violet, 20% methanol for 5 minutes. Nine fields per well were imaged at 100X magnification. The invasive cells in each field were counted using ImageJ software. Normalized invasion was calculated as a percentage compared to a control condition.

### Functional Inhibition of MMP-2

Cells were plated in 6 well plates and treated with either 10 μM Marimastat (EMD Millipore, Billerica, MA), 1 μM MMP-2 Inhibitor I (*Cis*-9-octadecenoyl-N-hydroxylamide oleoyl-N-hydroxylamide, EMD Millipore), or the equivalent volume of DMSO for 24 hours. An invasion assay was performed as described above. Cells were plated in six-well tissue culture plates and transfected per manufacturer's instructions with 20 μM MMP-2 siRNA or control siRNA with 3 μL of Dharamafect2 (Thermo Scientific, formerly Dharmacon) for 48 hours

### MTT Cell Growth Assays

Cells were seeded into a 96-well tissue culture plate at 2,4,6,8 and 10 × 10^3^ cells per well, in duplicate, to a total volume of 200 μL per well. Cells were incubated for 24 hours, then 20 μL of 5 mg/mL MTT (Sigma) was added to each well, and the plate was incubated for an additional 4 hours. Media was removed from each well, then 200 μL per well of DMSO was added and the plate was incubated for 5 minutes. The optical absorbance was measured at 550 nm.

### Cell Adhesion and Detachment Assays

Six-well tissue culture plates were pre-coated with collagen I, fibronectin, or matrigel (BD Biosciences, Bedford, MA) per manufacturer's directions. For cell attachment, 3 × 10^3^ cells were seeded on pre-coated plates (or directly on to non-coated tissue culture plastic). At the indicated time points, media and unattached cells were removed and the remaining attached cells were counted. For cell detachment assays, 5 × 10^4^ cells per well were seeded on pre-coated 12-well tissue culture plates. After 48 hours, cells were washed once with PBS, and 5% trypsin diluted in PBS was added to each well. Media was removed at the indicated time points and the number of detached cells were counted.

### Single Cell Motility Assays

A 35mm tissue culture dish (BD Falcon, Franklin Lakes, NJ) was pre-coated with 50 μg/mL collagen I (BD Biosciences, Bedford, MA) per maufacturer's directions. Cells were suspended in RPMI media with 10% FBS and 10,000 cells were added to the dish. Time-lapse images were taken every three minutes for 4.5 hours of seven randomly selected areas within the plate using a Biostation (Nikon Instruments, Melville, NY). The path traveled per cell was tracked for 5 cells per field using ImageJ software and the Manual Tracker plug-in.

### Authors' contributions

EAV and RCB conceived the study, planned experiments, wrote and revised the manuscript. EAV conducted the experiments. EAV and IMO conducted the orthotopic implantation mouse model. EAV, JMP and LX participated the generation of stable cell lines. EAV, IMO, JMP, XH, LX, BDJ, and RCB participated in data analysis. BJ participated in the design of the study and performed statistical analysis. All authors read and approved the final manuscript.

## SUPPLEMENTARY FIGURES


