# Supplementary material for: Heat shock protein 27 regulates human prostate cancer cell motility and metastatic progression
Source: Oncotarget. 2014 Apr 21;5(9):2648–63. doi: 10.18632/oncotarget.1917 (PMC4058034; doi:10.18632/oncotarget.1917)
Supplement: Supplementary file 1 [file oncotarget-05-2648-s001.pdf]

Heat shock protein 27 regulates human prostate cancer cell motility and metastatic progression

Additional File 1 - Supplemental Figures

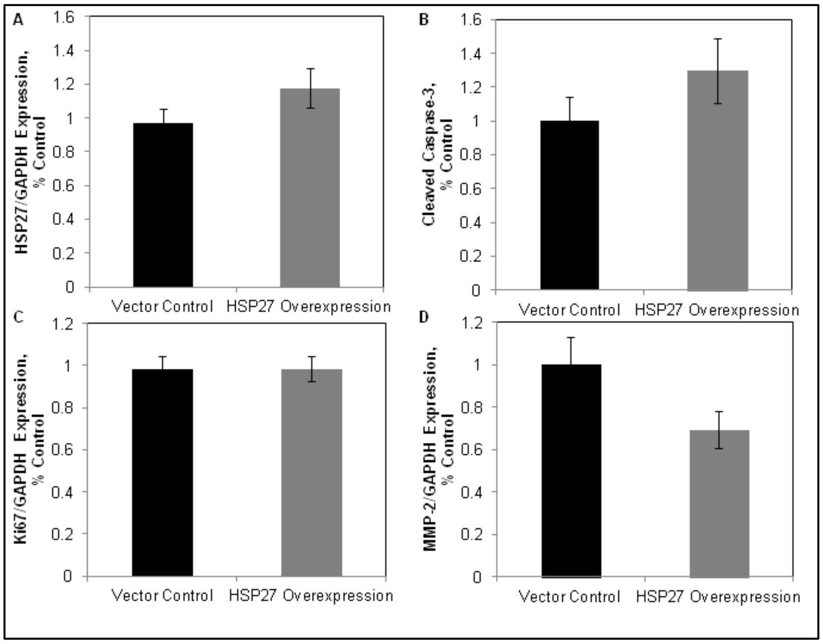

Figure S1: HSP27 gene expression was not altered at the conclusion of the six-week mouse experiment. Fresh frozen tumor tissue from the mice injected with HSP27 overexpression cells was analyzed for expression of HSP27 (A), Ki-67 (C) or MMP-2 (D) by qRT/PCR and for cleaved caspase-3 by ELISA (B). Data represent mean ± SEM for all mice in a given cohort. The cleaved caspase-3 concentration was measured in each tumor, in replicates of N=2 per mouse. Gene expression, normalized to GAPDH, was assessed in at least 2 independent experiments, each in replicates of N=2. All respective control values were normalized to 1.0. * denotes Student’s t-test $p \leq 0.05$ compared to controls.

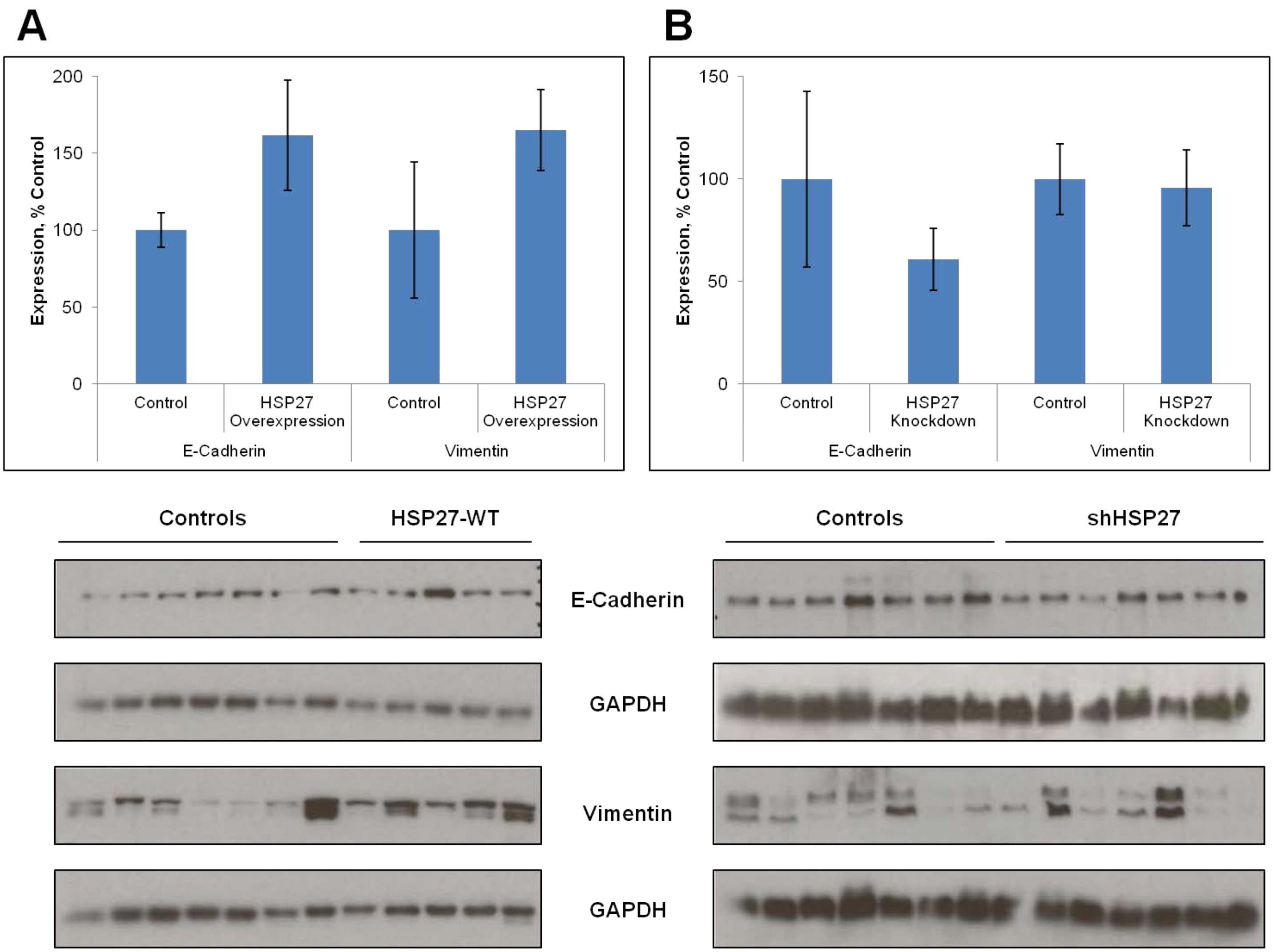

Figure S2: Protein expression levels of EMT markers in mouse xenograft tumors. Fresh frozen tumor tissue from mouse xenograft experiments was analyzed for expression of E-cadherin and vimentin by western blot. The graphs represent mean protein expression level of E-cadherin or vimentin $\pm$ SEM normalized to GAPDH and expressed relative to controls normalized to 100%.

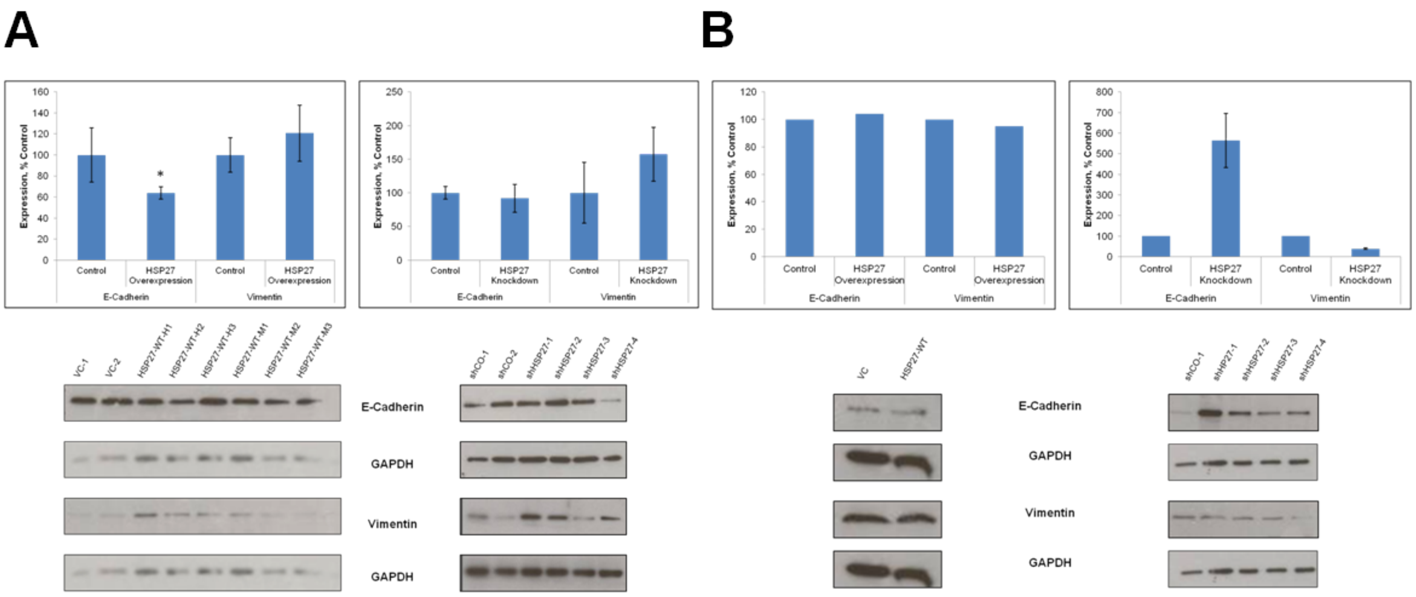

Figure S3: Protein expression levels of EMT markers in HSP27 stable variant cell lines. E-cadherin and vimentin protein levels were measured by Western blot in both PC3-M (A) and DU145 (B) derived stable cell lines. The graphs represent mean protein expression level $\pm$ SEM normalized to GAPDH and expressed relative to controls normalized to 100%. * denotes Student's t-test $p \leq 0.05$ compared to controls.

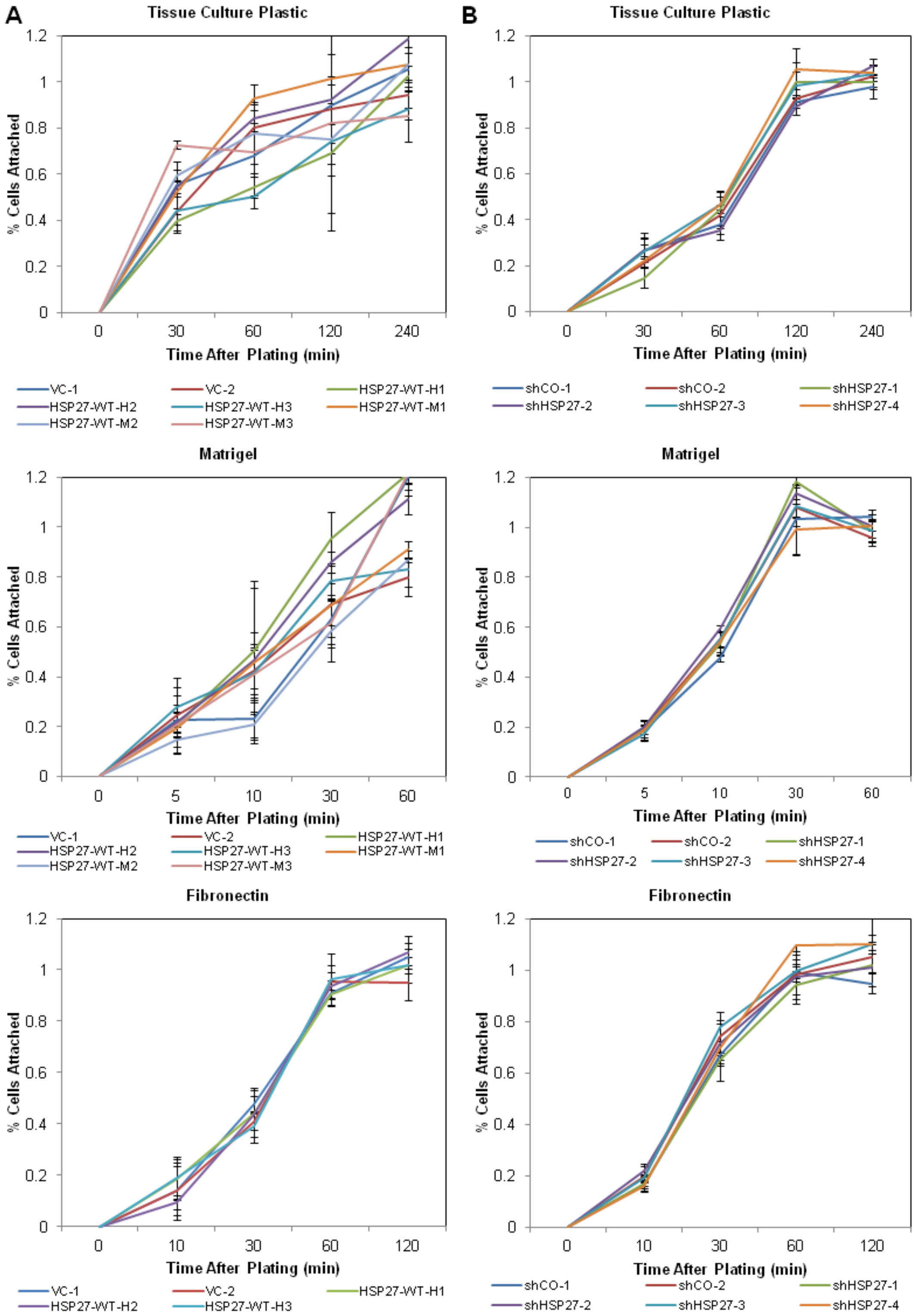

Figure S4: HSP27 does not alter cell attachment Cell attachment assays were performed as described in Methods. Data are the mean ± SEM attached cells with time after plating of the indicated overexpression (A) or shRNA knockdown cell lines (B). Attachment was measured using uncoated tissue culture plastic, Matrigel, and Fibronectin. Data is from a single experiment performed in replicates of N=3, with similar results seen in multiple separate experiments, also N=3.

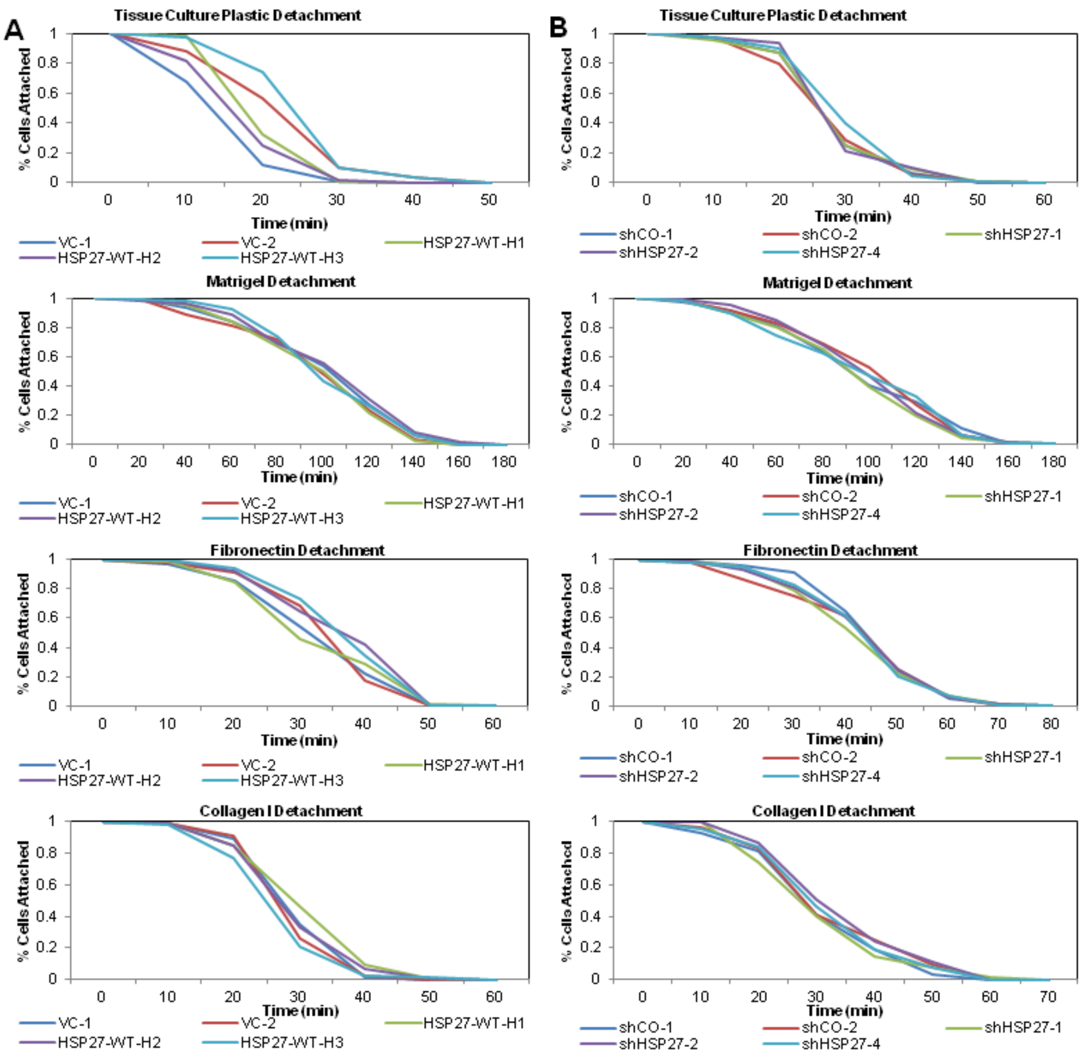

Figure S5: HSP27 does not alter cell detachment Cell detachment assays were performed as described in Methods. Data are the mean ± SEM remaing attached cells with time after addition of diluted trypsin of the indicated overexpression (A) or shRNA knockdown cell lines (B). Cell detachment was measured using uncoated tissue culture plastic, Matrigel, Fibronectin, and Collagen I. Data is from a single experiment performed in replicates of N=3, with similar results seen in multiple separate experiments, also N=3.

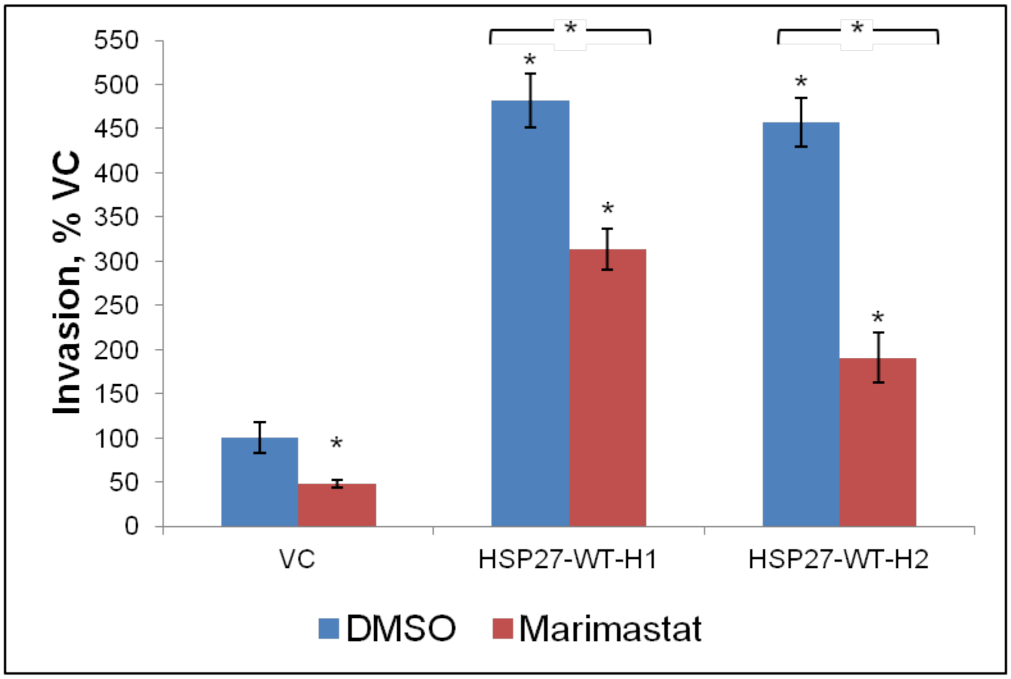

Figure S6: Broad MMP inhibition by Marimastat decreases cell invasion in HSP27 overexpressing cells

Cell invasion assays were performed as described in methods with PC3-M derived HSP27 overexpressing cell lines treated with 1 μM Marimastat or DMSO. Data represents mean cell invasion ± SEM expressed relative to DMSO-treated VC control cells normalized to 100%. The graph is from a single experiment with similar results observed in multiple experiments. * denotes Student's t-test $p \leq 0.05$ compared to DMSO-treated VC cells, or between the indicated groups.
